# 2-[(4-*tert*-Butyl­anilino)(phen­yl)meth­yl]cyclo­hexa­none

**DOI:** 10.1107/S1600536809004954

**Published:** 2009-02-18

**Authors:** Hoong-Kun Fun, Suchada Chantrapromma, Sankappa Rai, Prakash Shetty, Arun M. Isloor

**Affiliations:** a X-ray Crystallography Unit, School of Physics, Universiti Sains Malaysia, 11800 USM, Penang, Malaysia; b Crystal Materials Research Unit, Department of Chemistry, Faculty of Science, Prince of Songkla University, Hat-Yai, Songkhla 90112, Thailand; c Syngene International Ltd, Biocon Park, plot No. 2 & 3, Bommasandra 4^th^ Phase, Jigani Link Road, Bangalore 560 100, India; d Department of Printing, Manipal Institute of Technology, Manipal 576 104, India; e Department of Chemistry, National Institute of Technology - Karnataka, Surathkal, Mangalore 575 025, India

## Abstract

In the mol­ecule of the title compound, C_23_H_29_NO, the cyclo­hexa­none ring has been distorted from the standard chair conformation by the ketone group such that part of the ring is almost flat. The remaining [(4-*tert*-butyl­anilino)(phen­yl)meth­yl] portion of the mol­ecule is in an equatorial position on the cyclo­hexa­none ring. The dihedral angle between the two benzene rings is 81.52 (8)°. In the crystal packing, mol­ecules are linked by N—H⋯O hydrogen bonds into infinite one-dimensional chains along the *a* axis and these chains are stacked down the *c* axis. The crystal structure is further stabilized by weak C—H⋯O and C—H⋯π inter­actions.

## Related literature

For values of bond lengths, see: Allen *et al.* (1987 ▶). For details of hydrogen-bond motifs, see: Bernstein *et al.* (1995 ▶). For information on the Mannich reaction, see: Kobayashi & Ishitani (1999 ▶); Bohme & Haake (1976 ▶). For background to the bioactivity and applications of beta-amino carbonyl compounds, see, for example: Arend *et al.* (1988 ▶); Isloor, Sunil *et al.* (2009 ▶); Isloor, Kalluraya *et al.* (2009 ▶); Jadhav *et al.* (2008 ▶); Kalluraya *et al.* (2001 ▶). For puckering parameters, see: Cremer & Pople, (1975 ▶). For the stability of the temperature controller, see Cosier & Glazer (1986 ▶).
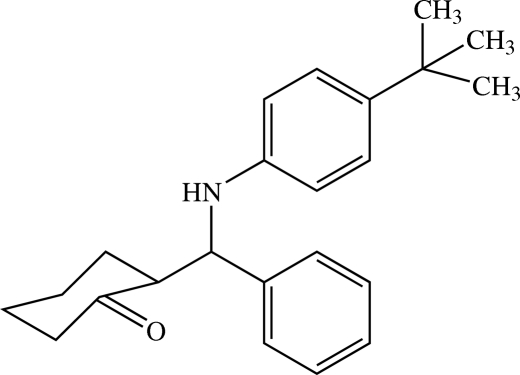


## Experimental

### 

#### Crystal data

C_23_H_29_NO
*M* *_r_* = 335.47
Triclinic, 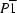

*a* = 6.5315 (2) Å
*b* = 12.3946 (3) Å
*c* = 12.8853 (3) Å
α = 62.973 (1)°
β = 86.347 (2)°
γ = 85.103 (2)°
*V* = 925.46 (4) Å^3^
*Z* = 2
Mo *K*α radiation
μ = 0.07 mm^−1^
*T* = 100 K
0.52 × 0.41 × 0.11 mm

#### Data collection

Bruker SMART APEXII CCD area-detector diffractometer
Absorption correction: multi-scan (*SADABS*; Bruker, 2005 ▶) *T*_min_ = 0.953, *T*_max_ = 0.992
17722 measured reflections
4449 independent reflections
3584 reflections with *I* > 2σ(*I*)
*R*_int_ = 0.027

#### Refinement

*R*[*F* ^2^ > 2σ(*F* ^2^)] = 0.047
*wR*(*F* ^2^) = 0.124
*S* = 1.07
4449 reflections
233 parameters
H atoms treated by a mixture of independent and constrained refinement
Δρ_max_ = 0.39 e Å^−3^
Δρ_min_ = −0.23 e Å^−3^

### 

Data collection: *APEX2* (Bruker, 2005 ▶); cell refinement: *APEX2*; data reduction: *SAINT* (Bruker, 2005 ▶); program(s) used to solve structure: *SHELXTL* (Sheldrick, 2008 ▶); program(s) used to refine structure: *SHELXTL*; molecular graphics: *SHELXTL*; software used to prepare material for publication: *SHELXTL* and *PLATON* (Spek, 2009 ▶).

## Supplementary Material

Crystal structure: contains datablocks global, I. DOI: 10.1107/S1600536809004954/sj2573sup1.cif

Structure factors: contains datablocks I. DOI: 10.1107/S1600536809004954/sj2573Isup2.hkl

Additional supplementary materials:  crystallographic information; 3D view; checkCIF report

## Figures and Tables

**Table 1:** Hydrogen-bond geometry (Å, °)

*D*—H⋯*A*	*D*—H	H⋯*A*	*D*⋯*A*	*D*—H⋯*A*
N1—H1*N*1⋯O1^i^	0.89 (2)	2.35 (2)	3.2050 (16)	161.6 (19)
C9—H9*A*⋯O1	0.93	2.59	3.1146 (19)	116
C2—H2*A*⋯*Cg*1^ii^	0.97	2.60	3.4992 (19)	155
C23—H23*C*⋯*Cg*1^iii^	0.96	2.99	3.747 (2)	137

Symmetry codes: (i) 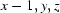; (ii) 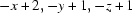; (iii) 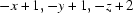. *Cg*1 is the centroid of the C14–C19 ring.

**Fig. 1.**
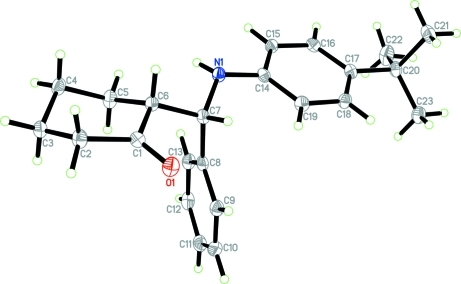
The structure of (I), showing 50% probability displacement ellipsoids and the atom-numbering scheme.

**Fig. 2.**
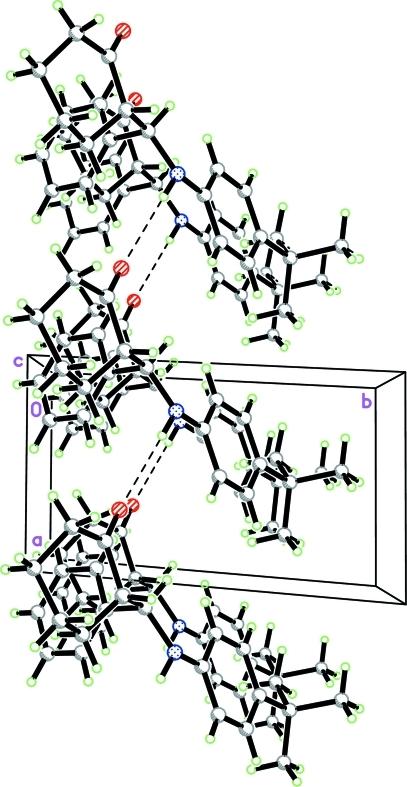
The packing diagram of (I), viewed along the c axis, showing molecular chains along the a axis. Hydrogen bonds are shown as dashed lines.

## supplementary crystallographic information

### Comment

Mannich reactions are among the most important carbon-carbon bond forming reactions in organic synthesis (Kobayashi & Ishitani, 1999). They provide beta-amino carbonyl compounds, which are important synthetic intermediates for various pharmaceuticals and natural products (Arend *et al.*, 1988). They exhibit wide variety of pharmaceutical properties such as anti cancer (Isloor, Sunil *et al.*, 2009), analgesic (Isloor, Kalluraya *et al.*, 2009), anti-inflammatory (Jadhav *et al.*, 2008), antimicrobial (Kalluraya *et al.*, 2001) activities. The increasing popularity of the Mannich reaction has been fueled by the ubiquitous nature of nitrogen containing compounds in drugs and natural products (Bohme & Haake, 1976). Prompted by the biological activity of these derivatives, we have synthesized the title compound (I) and report its structure here, Fig 1.  The cyclohexanone ring has been distorted from the standard chair conformation by the ketone group such that the C2 C1 O1 C6 part of the ring is almost flat with puckering parameter Q = 0.5181 (16)Å, and θ = 159.23 (18)° and φ = 9.3 (5)° (Cremer & Pople, 1975). The [4-(*tert*-butyl)anilino](phenyl)methyl substituent group is equatorially attached to the ring at atom C6 with torsion angles C5–C6–C7–C8 = -57.80 (16)° and C5–C6–C7–N1 = 69.13 (15)°. The two benzene rings are nearly perpendicular to each other with a  dihedral angle of  81.52 (8)° between them. The bond distances have normal values (Allen *et al.*, 1987).

A  weak intramolecular C9—H9A···O1 interaction generates an S(7) ring motif (Bernstein *et al.*, 1995) (Table 1) and effects the solid state conformation of the molecule. In the crystal structure N—H···O hydrogen bonds (Table 1, Fig. 2) link the molecules into infinite one-dimensional chains along the *a* axis and these chains are stacked down the *c* axis. The crystal is further stabilized by weak C—H···O and C—H···π interactions (Table 1); *Cg*1 is the centroid of the C14–C19 ring (Table 1).

### Experimental

The title compound was obtained by vigorously stirring a solution of cyclohexanone (0.5 g, 5.0 mmol), benzaldehyde (0.53 g, 5.0 mmol) and 4-*tert*-butylaniline (0.75 g, 5.0 mmol) in dry acetonitrile (5 ml). Trifluoro acetic acid (0.57 g, 5 mmol) was then added. The reaction mixture was stirred at room temperature for 2 h. After standing for 1 hr, the solvent was removed and the crude product was purified by column chromatography using ethyl acetate and petroleum ether (1:1 *v*:*v*) as eluants. The product was further recrystalized using 10 ml of hot ethanol. The yield was 1 g (58%), *M*.p 439–441 K.

### Refinement

The amine H atom was located in a difference map and refined isotropically. The remaining H atoms were placed in calculated positions with d(C—H) = 0.93 Å, *U*_iso_=1.2*U*_eq_(C) for aromatic, 0.98 Å, *U*_iso_=1.2*U*_eq_(C) for CH, 0.97 Å, *U*_iso_=1.2*U*_eq_(C) for CH_2_ and 0.96 Å, *U*_iso_ = 1.5*U*_eq_(C) for CH_3_ atoms. A rotating group model was used for the methyl groups. The highest residual electron density peak is located at 0.72 Å from C8 and the deepest hole is located at 1.03 Å from C16.

### Crystal data

**Table:**

C_23_H_29_NO	*Z* = 2
*M**_r_* = 335.47	*F*(000) = 364
Triclinic, *P*1	*D*_x_ = 1.204 Mg m^−^^3^
Hall symbol: -P 1	Melting point = 439–441 K
*a* = 6.5315 (2) Å	Mo *K*α radiation, λ = 0.71073 Å
*b* = 12.3946 (3) Å	Cell parameters from 4449 reflections
*c* = 12.8853 (3) Å	θ = 1.8–28.0°
α = 62.973 (1)°	µ = 0.07 mm^−^^1^
β = 86.347 (2)°	*T* = 100 K
γ = 85.103 (2)°	Plate, colorless
*V* = 925.46 (4) Å^3^	0.52 × 0.41 × 0.11 mm

### Data collection

**Table:**

Bruker SMART APEXII CCD area-detector diffractometer	4449 independent reflections
Radiation source: fine-focus sealed tube	3584 reflections with *I* > 2σ(*I*)
graphite	*R*_int_ = 0.027
Detector resolution: 8.33 pixels mm^-1^	θ_max_ = 28.0°, θ_min_ = 1.8°
ω scans	*h* = −8→8
Absorption correction: multi-scan (*SADABS*; Bruker, 2005)	*k* = −16→16
*T*_min_ = 0.953, *T*_max_ = 0.992	*l* = −16→16
17722 measured reflections	

### Refinement

**Table:**

Refinement on *F*^2^	Primary atom site location: structure-invariant direct methods
Least-squares matrix: full	Secondary atom site location: difference Fourier map
*R*[*F*^2^ > 2σ(*F*^2^)] = 0.047	Hydrogen site location: inferred from neighbouring sites
*wR*(*F*^2^) = 0.124	H atoms treated by a mixture of independent and constrained refinement
*S* = 1.07	*w* = 1/[σ^2^(*F*_o_^2^) + (0.0465*P*)^2^ + 0.607*P*] where *P* = (*F*_o_^2^ + 2*F*_c_^2^)/3
4449 reflections	(Δ/σ)_max_ < 0.001
233 parameters	Δρ_max_ = 0.39 e Å^−^^3^
0 restraints	Δρ_min_ = −0.23 e Å^−^^3^

### Special details

**Table:**

Experimental. The crystal was placed in the cold stream of an Oxford Cryosystems Cobra open-flow nitrogen cryostat (Cosier & Glazer, 1986) operating at 100.0 (1) K.
Geometry. All e.s.d.'s (except the e.s.d. in the dihedral angle between two l.s. planes) are estimated using the full covariance matrix. The cell e.s.d.'s are taken into account individually in the estimation of e.s.d.'s in distances, angles and torsion angles; correlations between e.s.d.'s in cell parameters are only used when they are defined by crystal symmetry. An approximate (isotropic) treatment of cell e.s.d.'s is used for estimating e.s.d.'s involving l.s. planes.
Refinement. Refinement of *F*^2^ against ALL reflections. The weighted *R*-factor *wR* and goodness of fit *S* are based on *F*^2^, conventional *R*-factors *R* are based on *F*, with *F* set to zero for negative *F*^2^. The threshold expression of *F*^2^ > σ(*F*^2^) is used only for calculating *R*-factors(gt) *etc*. and is not relevant to the choice of reflections for refinement. *R*-factors based on *F*^2^ are statistically about twice as large as those based on *F*, and *R*- factors based on ALL data will be even larger.

### Fractional atomic coordinates and isotropic or equivalent isotropic displacement parameters (Å^2^)

**Table:**

	*x*	*y*	*z*	*U*_iso_*/*U*_eq_	
O1	1.32798 (15)	0.71893 (10)	0.43723 (9)	0.0200 (2)	
N1	0.75282 (19)	0.58289 (11)	0.55557 (10)	0.0157 (3)	
C1	1.1976 (2)	0.75037 (12)	0.36390 (12)	0.0147 (3)	
C2	1.2523 (2)	0.82454 (13)	0.23594 (12)	0.0183 (3)	
H2A	1.2974	0.7691	0.2037	0.022*	
H2B	1.3680	0.8714	0.2299	0.022*	
C3	1.0809 (2)	0.91152 (13)	0.16072 (12)	0.0176 (3)	
H3A	1.0622	0.9816	0.1759	0.021*	
H3B	1.1192	0.9396	0.0790	0.021*	
C4	0.8804 (2)	0.84862 (14)	0.18722 (12)	0.0189 (3)	
H4A	0.8968	0.7809	0.1684	0.023*	
H4B	0.7727	0.9051	0.1396	0.023*	
C5	0.8193 (2)	0.80271 (14)	0.31607 (12)	0.0194 (3)	
H5A	0.6875	0.7670	0.3308	0.023*	
H5B	0.8042	0.8707	0.3346	0.023*	
C6	0.9799 (2)	0.70785 (12)	0.39536 (12)	0.0146 (3)	
H6A	0.9781	0.6384	0.3785	0.018*	
C7	0.9298 (2)	0.65755 (12)	0.52741 (12)	0.0144 (3)	
H7A	1.0474	0.6032	0.5682	0.017*	
C8	0.9016 (2)	0.75555 (12)	0.56920 (12)	0.0153 (3)	
C9	1.0660 (2)	0.78409 (13)	0.61390 (12)	0.0183 (3)	
H9A	1.1930	0.7419	0.6200	0.022*	
C10	1.0424 (3)	0.87519 (14)	0.64956 (13)	0.0220 (3)	
H10A	1.1535	0.8941	0.6785	0.026*	
C11	0.8531 (3)	0.93747 (14)	0.64179 (13)	0.0233 (3)	
H11A	0.8377	0.9994	0.6641	0.028*	
C12	0.6871 (2)	0.90756 (14)	0.60086 (13)	0.0216 (3)	
H12A	0.5592	0.9480	0.5977	0.026*	
C13	0.7104 (2)	0.81741 (13)	0.56449 (12)	0.0184 (3)	
H13A	0.5980	0.7981	0.5368	0.022*	
C14	0.6904 (2)	0.51586 (12)	0.67217 (12)	0.0147 (3)	
C15	0.4914 (2)	0.47478 (13)	0.69928 (12)	0.0159 (3)	
H15A	0.4004	0.4953	0.6396	0.019*	
C16	0.4279 (2)	0.40402 (12)	0.81375 (12)	0.0165 (3)	
H16A	0.2945	0.3788	0.8288	0.020*	
C17	0.5577 (2)	0.36937 (12)	0.90732 (12)	0.0159 (3)	
C18	0.7558 (2)	0.41071 (13)	0.87917 (12)	0.0173 (3)	
H18A	0.8472	0.3892	0.9389	0.021*	
C19	0.8215 (2)	0.48285 (13)	0.76503 (12)	0.0172 (3)	
H19A	0.9541	0.5094	0.7503	0.021*	
C20	0.4898 (2)	0.28876 (13)	1.03410 (12)	0.0178 (3)	
C21	0.5531 (3)	0.15560 (14)	1.06508 (14)	0.0280 (4)	
H21A	0.4873	0.1327	1.0142	0.042*	
H21B	0.5121	0.1048	1.1444	0.042*	
H21C	0.6996	0.1461	1.0560	0.042*	
C22	0.2558 (2)	0.30252 (16)	1.05089 (14)	0.0277 (4)	
H22A	0.1889	0.2703	1.0078	0.042*	
H22B	0.2133	0.3869	1.0233	0.042*	
H22C	0.2191	0.2590	1.1321	0.042*	
C23	0.5907 (3)	0.32236 (15)	1.11896 (13)	0.0235 (3)	
H23A	0.7367	0.3055	1.1169	0.035*	
H23B	0.5370	0.2754	1.1965	0.035*	
H23C	0.5616	0.4072	1.0966	0.035*	
H1N1	0.646 (3)	0.6162 (18)	0.5096 (17)	0.030 (5)*	

### Atomic displacement parameters (Å^2^)

**Table:**

	*U*^11^	*U*^22^	*U*^33^	*U*^12^	*U*^13^	*U*^23^
O1	0.0126 (5)	0.0257 (6)	0.0188 (5)	−0.0004 (4)	−0.0006 (4)	−0.0076 (4)
N1	0.0128 (6)	0.0171 (6)	0.0142 (6)	−0.0028 (5)	−0.0003 (5)	−0.0043 (5)
C1	0.0119 (6)	0.0148 (6)	0.0168 (7)	0.0009 (5)	0.0008 (5)	−0.0072 (5)
C2	0.0135 (7)	0.0216 (7)	0.0163 (7)	−0.0015 (5)	0.0028 (5)	−0.0059 (6)
C3	0.0182 (7)	0.0177 (7)	0.0140 (7)	−0.0012 (5)	0.0004 (5)	−0.0048 (6)
C4	0.0169 (7)	0.0221 (7)	0.0150 (7)	−0.0011 (6)	−0.0028 (5)	−0.0058 (6)
C5	0.0121 (7)	0.0243 (8)	0.0161 (7)	−0.0004 (5)	0.0000 (5)	−0.0044 (6)
C6	0.0121 (6)	0.0158 (6)	0.0147 (7)	−0.0012 (5)	−0.0003 (5)	−0.0057 (5)
C7	0.0110 (6)	0.0150 (6)	0.0140 (6)	−0.0008 (5)	−0.0001 (5)	−0.0039 (5)
C8	0.0159 (7)	0.0151 (6)	0.0117 (6)	−0.0024 (5)	0.0017 (5)	−0.0032 (5)
C9	0.0155 (7)	0.0205 (7)	0.0153 (7)	−0.0028 (5)	0.0007 (5)	−0.0048 (6)
C10	0.0262 (8)	0.0234 (8)	0.0158 (7)	−0.0079 (6)	−0.0007 (6)	−0.0074 (6)
C11	0.0362 (9)	0.0175 (7)	0.0152 (7)	−0.0017 (6)	0.0016 (6)	−0.0067 (6)
C12	0.0236 (8)	0.0206 (7)	0.0161 (7)	0.0040 (6)	0.0006 (6)	−0.0054 (6)
C13	0.0162 (7)	0.0207 (7)	0.0151 (7)	−0.0008 (5)	0.0001 (5)	−0.0056 (6)
C14	0.0142 (7)	0.0125 (6)	0.0156 (7)	0.0002 (5)	0.0012 (5)	−0.0051 (5)
C15	0.0137 (7)	0.0160 (7)	0.0163 (7)	−0.0005 (5)	−0.0025 (5)	−0.0058 (5)
C16	0.0126 (6)	0.0156 (7)	0.0199 (7)	−0.0018 (5)	0.0014 (5)	−0.0068 (6)
C17	0.0160 (7)	0.0138 (6)	0.0157 (7)	0.0006 (5)	0.0021 (5)	−0.0054 (5)
C18	0.0148 (7)	0.0189 (7)	0.0162 (7)	0.0010 (5)	−0.0027 (5)	−0.0063 (6)
C19	0.0116 (6)	0.0193 (7)	0.0187 (7)	−0.0016 (5)	0.0006 (5)	−0.0070 (6)
C20	0.0168 (7)	0.0176 (7)	0.0150 (7)	−0.0005 (5)	0.0018 (5)	−0.0042 (6)
C21	0.0387 (10)	0.0179 (7)	0.0216 (8)	−0.0027 (7)	0.0058 (7)	−0.0046 (6)
C22	0.0184 (8)	0.0376 (9)	0.0187 (8)	−0.0029 (7)	0.0047 (6)	−0.0059 (7)
C23	0.0262 (8)	0.0249 (8)	0.0168 (7)	−0.0020 (6)	0.0009 (6)	−0.0072 (6)

### Geometric parameters (Å, °)

**Table:**

O1—C1	1.2172 (17)	C11—C12	1.382 (2)
N1—C14	1.3996 (18)	C11—H11A	0.9300
N1—C7	1.4657 (17)	C12—C13	1.389 (2)
N1—H1N1	0.89 (2)	C12—H12A	0.9300
C1—C2	1.5149 (19)	C13—H13A	0.9300
C1—C6	1.5244 (19)	C14—C19	1.399 (2)
C2—C3	1.529 (2)	C14—C15	1.4019 (19)
C2—H2A	0.9700	C15—C16	1.388 (2)
C2—H2B	0.9700	C15—H15A	0.9300
C3—C4	1.521 (2)	C16—C17	1.398 (2)
C3—H3A	0.9700	C16—H16A	0.9300
C3—H3B	0.9700	C17—C18	1.397 (2)
C4—C5	1.5281 (19)	C17—C20	1.5376 (19)
C4—H4A	0.9700	C18—C19	1.392 (2)
C4—H4B	0.9700	C18—H18A	0.9300
C5—C6	1.537 (2)	C19—H19A	0.9300
C5—H5A	0.9700	C20—C23	1.535 (2)
C5—H5B	0.9700	C20—C22	1.535 (2)
C6—C7	1.5462 (18)	C20—C21	1.536 (2)
C6—H6A	0.9800	C21—H21A	0.9600
C7—C8	1.5307 (19)	C21—H21B	0.9600
C7—H7A	0.9800	C21—H21C	0.9600
C8—C9	1.392 (2)	C22—H22A	0.9600
C8—C13	1.398 (2)	C22—H22B	0.9600
C9—C10	1.394 (2)	C22—H22C	0.9600
C9—H9A	0.9300	C23—H23A	0.9600
C10—C11	1.384 (2)	C23—H23B	0.9600
C10—H10A	0.9300	C23—H23C	0.9600
			
C14—N1—C7	119.50 (12)	C12—C11—C10	119.94 (14)
C14—N1—H1N1	111.8 (13)	C12—C11—H11A	120.0
C7—N1—H1N1	115.8 (13)	C10—C11—H11A	120.0
O1—C1—C2	120.57 (12)	C11—C12—C13	120.31 (14)
O1—C1—C6	121.72 (12)	C11—C12—H12A	119.8
C2—C1—C6	117.49 (12)	C13—C12—H12A	119.8
C1—C2—C3	116.04 (12)	C12—C13—C8	120.49 (14)
C1—C2—H2A	108.3	C12—C13—H13A	119.8
C3—C2—H2A	108.3	C8—C13—H13A	119.8
C1—C2—H2B	108.3	C19—C14—N1	122.84 (13)
C3—C2—H2B	108.3	C19—C14—C15	117.33 (12)
H2A—C2—H2B	107.4	N1—C14—C15	119.78 (13)
C4—C3—C2	110.53 (12)	C16—C15—C14	121.08 (13)
C4—C3—H3A	109.5	C16—C15—H15A	119.5
C2—C3—H3A	109.5	C14—C15—H15A	119.5
C4—C3—H3B	109.5	C15—C16—C17	122.24 (13)
C2—C3—H3B	109.5	C15—C16—H16A	118.9
H3A—C3—H3B	108.1	C17—C16—H16A	118.9
C3—C4—C5	110.11 (12)	C18—C17—C16	116.14 (13)
C3—C4—H4A	109.6	C18—C17—C20	121.38 (13)
C5—C4—H4A	109.6	C16—C17—C20	122.47 (13)
C3—C4—H4B	109.6	C19—C18—C17	122.45 (13)
C5—C4—H4B	109.6	C19—C18—H18A	118.8
H4A—C4—H4B	108.2	C17—C18—H18A	118.8
C4—C5—C6	111.74 (12)	C18—C19—C14	120.75 (13)
C4—C5—H5A	109.3	C18—C19—H19A	119.6
C6—C5—H5A	109.3	C14—C19—H19A	119.6
C4—C5—H5B	109.3	C23—C20—C22	107.70 (13)
C6—C5—H5B	109.3	C23—C20—C21	108.66 (13)
H5A—C5—H5B	107.9	C22—C20—C21	109.07 (13)
C1—C6—C5	112.30 (11)	C23—C20—C17	111.26 (12)
C1—C6—C7	112.15 (11)	C22—C20—C17	111.07 (12)
C5—C6—C7	114.88 (11)	C21—C20—C17	109.02 (12)
C1—C6—H6A	105.5	C20—C21—H21A	109.5
C5—C6—H6A	105.5	C20—C21—H21B	109.5
C7—C6—H6A	105.5	H21A—C21—H21B	109.5
N1—C7—C8	113.40 (11)	C20—C21—H21C	109.5
N1—C7—C6	108.22 (11)	H21A—C21—H21C	109.5
C8—C7—C6	113.68 (11)	H21B—C21—H21C	109.5
N1—C7—H7A	107.1	C20—C22—H22A	109.5
C8—C7—H7A	107.1	C20—C22—H22B	109.5
C6—C7—H7A	107.1	H22A—C22—H22B	109.5
C9—C8—C13	118.57 (13)	C20—C22—H22C	109.5
C9—C8—C7	120.52 (13)	H22A—C22—H22C	109.5
C13—C8—C7	120.91 (13)	H22B—C22—H22C	109.5
C8—C9—C10	120.75 (14)	C20—C23—H23A	109.5
C8—C9—H9A	119.6	C20—C23—H23B	109.5
C10—C9—H9A	119.6	H23A—C23—H23B	109.5
C11—C10—C9	119.90 (14)	C20—C23—H23C	109.5
C11—C10—H10A	120.1	H23A—C23—H23C	109.5
C9—C10—H10A	120.1	H23B—C23—H23C	109.5
			
O1—C1—C2—C3	−149.78 (14)	C9—C10—C11—C12	1.3 (2)
C6—C1—C2—C3	35.51 (18)	C10—C11—C12—C13	−1.7 (2)
C1—C2—C3—C4	−45.58 (17)	C11—C12—C13—C8	0.2 (2)
C2—C3—C4—C5	58.62 (16)	C9—C8—C13—C12	1.7 (2)
C3—C4—C5—C6	−62.20 (16)	C7—C8—C13—C12	−178.96 (13)
O1—C1—C6—C5	148.61 (14)	C7—N1—C14—C19	−22.0 (2)
C2—C1—C6—C5	−36.74 (17)	C7—N1—C14—C15	160.68 (12)
O1—C1—C6—C7	17.48 (18)	C19—C14—C15—C16	−0.1 (2)
C2—C1—C6—C7	−167.88 (12)	N1—C14—C15—C16	177.38 (13)
C4—C5—C6—C1	49.83 (16)	C14—C15—C16—C17	−0.5 (2)
C4—C5—C6—C7	179.57 (12)	C15—C16—C17—C18	0.3 (2)
C14—N1—C7—C8	−59.87 (16)	C15—C16—C17—C20	−178.69 (13)
C14—N1—C7—C6	173.04 (12)	C16—C17—C18—C19	0.4 (2)
C1—C6—C7—N1	−161.06 (11)	C20—C17—C18—C19	179.42 (13)
C5—C6—C7—N1	69.13 (15)	C17—C18—C19—C14	−1.0 (2)
C1—C6—C7—C8	72.02 (15)	N1—C14—C19—C18	−176.59 (13)
C5—C6—C7—C8	−57.80 (16)	C15—C14—C19—C18	0.8 (2)
N1—C7—C8—C9	141.87 (13)	C18—C17—C20—C23	34.25 (18)
C6—C7—C8—C9	−93.95 (15)	C16—C17—C20—C23	−146.77 (14)
N1—C7—C8—C13	−37.48 (17)	C18—C17—C20—C22	154.21 (14)
C6—C7—C8—C13	86.69 (15)	C16—C17—C20—C22	−26.80 (19)
C13—C8—C9—C10	−2.1 (2)	C18—C17—C20—C21	−85.57 (17)
C7—C8—C9—C10	178.51 (12)	C16—C17—C20—C21	93.41 (17)
C8—C9—C10—C11	0.7 (2)		

### Hydrogen-bond geometry (Å, °)

**Table:**

*D*—H···*A*	*D*—H	H···*A*	*D*···*A*	*D*—H···*A*
N1—H1N1···O1^i^	0.89 (2)	2.35 (2)	3.2050 (16)	161.6 (19)
C9—H9A···O1	0.93	2.59	3.1146 (19)	116
C2—H2A···Cg1^ii^	0.97	2.60	3.4992 (19)	155
C23—H23C···Cg1^iii^	0.96	2.99	3.747 (2)	137

Symmetry codes: (i) *x*−1, *y*, *z*;  (ii) −*x*+2, −*y*+1, −*z*+1; (iii) −*x*+1, −*y*+1, −*z*+2.
